# Cost-utility and budget impact analysis of herpes zoster vaccines in Thai patients with end-stage renal disease

**DOI:** 10.1080/20523211.2026.2701918

**Published:** 2026-07-23

**Authors:** Nattanichcha Kulthanachairojana, Pattheera Phothong, Pitch Promkladphanao, Chollakarn Singharerg, Jatapat Hemapanpairoa

**Affiliations:** a Department of Social and Administrative Pharmacy, Faculty of Pharmaceutical Sciences, Burapha University, Chonburi, Thailand; b Faculty of Pharmaceutical Sciences, Burapha University, Chonburi, Thailand; c Division of Pharmaceutical Care, Faculty of Pharmacy, Silpakorn University, Nakhon Pathom, Thailand

**Keywords:** Cost-utility analysis, end-stage renal disease, herpes zoster, Thailand, vaccine

## Abstract

**Background:**

Patients with end-stage renal disease (ESRD) are increased risk of herpes zoster (HZ). The economic value of HZ vaccines in Thai patients with ESRD has not been assessed. This study evaluated the cost-utility and budget impact of two HZ vaccination strategies – zoster vaccine live (ZVL) and recombinant zoster vaccine (RZV) – compared with no vaccination in Thailand.

**Methods:**

A Markov model was developed to estimate lifetime health and economic outcomes in patients with ESRD. Cost-utility analysis was conducted from a societal perspective, while budget impact analysis was performed from a payer perspective over a 5-year time horizon. Model inputs were derived from published literature and Thai data sources. Uncertainty was assessed using deterministic, probabilistic, and scenario sensitivity analyses.

**Results:**

In the base-case analysis, both ZVL and RZV were cost-effective compared with no vaccination, with incremental cost-effectiveness ratios (ICER) of USD 1,599.14 (THB 51,912.56) and USD 2,920.09 (THB 94,794.30) per quality-adjusted life year (QALY) gained, respectively – both below Thailand’s willingness-to-pay threshold. RZV generated greater health benefits but was associated with higher costs. Sensitivity analyses confirmed the robustness of cost-effectiveness results across key assumptions. Over a 5-year period, the estimated budget impact ranged from USD 4.99–9.55 million (THB 161.86–310.02 million) for ZVL and USD 28.92–55.42 million (THB 938.95–1,799.09 million) for RZV, depending on vaccine uptake assumptions, with expenditures largely concentrated in the first year due to vaccination of prevalent patients.

**Conclusion:**

Both HZ vaccines are cost-effective options for preventing HZ in Thai patients with ESRD. While RZV provides greater health gains, it requires substantially higher budgetary investment. These findings support prioritising HZ vaccination for patients with ESRD and initiating pilot implementation within dialysis care settings to assess system-level impact and feasibility.

## Key learning points

**What was known:**

- Patients with end-stage renal disease (ESRD) face a higher risk of developing herpes zoster (HZ).
- Despite HZ vaccine availability, uptake in Thailand remains limited.
- No prior study has evaluated the cost-effectiveness of HZ vaccination specifically in Thai patients with ESRD.

**This study adds:**

- This study is the first economic assessment of HZ vaccination specifically in Thai ESRD patients.
- Both HZ vaccine strategies were found to be cost-effective compared to no vaccination.
- RZV provided the greatest clinical benefit in terms of reducing HZ and its complications.

**Potential impact:**

- This study provides evidence to support vaccination policies for HZ prevention in high-risk populations with renal disease.
- Our findings offer an economic rationale for introducing RZV into dialysis care pathways and may inform future reimbursement decisions and national vaccine prioritisation efforts in Thailand.

## Background

Herpes zoster (HZ) is a painful and often disabling condition caused by reactivation of the varicella zoster virus, which lies dormant in sensory ganglia after the initial infection. The disease mainly affects older adults and is associated with considerable morbidity (Lim et al., 2024). One of its most common complications is post-herpetic neuralgia (PHN). This chronic neuropathic pain may last for months or even years after the rash resolves, severely disrupting sleep, mobility, and daily activities (Drolet et al., 2010; Saguil et al., 2017). Other serious consequences include herpes zoster ophthalmicus (HZO), neurological complications, and disseminated infection, which in severe cases may require hospitalisation (Yawn & Gilden, 2013).

The burden of HZ is particularly pronounced among high-risk populations, with patients with end-stage renal disease (ESRD) representing one of the most vulnerable groups (Kuo et al., 2012). In these patients, impaired immune function, multiple co-existing conditions, and frequent exposure to healthcare settings increase the risk of developing both the infection and severe complications associated with it (Centers for Disease Control and Prevention, 2023). In Thailand, 110,764 patients were receiving dialysis in 2022 (Satirapoj et al., 2025). Compared with the general population, ESRD patients have a much higher incidence of HZ and are at a higher risk of mortality when infected (Ahn et al., 2019). In addition to personal suffering, HZ in dialysis patients can interrupt treatment schedules, cause problems related to infection control within dialysis units, and add strain to an already stretched healthcare system (Centers for Disease Control and Prevention, 2023).

Two vaccines are available in Thailand for HZ prevention: a live-attenuated vaccine (ZVL) and a recombinant subunit vaccine (RZV). Both have been shown to reduce the risk of HZ and its complications (Ansaldi et al., 2016; Williams et al., 2025). While ZVL is contraindicated in severely immunocompromised individuals (Ansaldi et al., 2016), RZV can be administered to a broader range of patients and is recommended in several high-income countries for high-risk adult populations, including patients suffering from chronic kidney disease (Advisory Committee on Immunization Practices, 2024). Despite these benefits, neither vaccine is included in Thailand’s national immunisation programme, and there are no specific recommendations for ESRD patients.

Internationally, vaccine policy decisions are informed by clinical benefit and economic value. National Immunization Technical Advisory Groups – such as the Advisory Committee on Immunization Practices (United States), the Joint Committee on Vaccination and Immunisation (United Kingdom), the National Advisory Committee on Immunization (Canada), and the WHO Strategic Advisory Group of Experts on Immunization (Government of, 2022; Matanock et al., 2020; Public Health, 2013; World Health, 2024) – require that the cost-effectiveness of vaccines be considered alongside safety and efficacy. Similarly, in Thailand, vaccine inclusion within national health benefit packages is guided by health technology assessment (HTA), in which cost-effectiveness and budget impact evidence generated by the Health Intervention and Technology Assessment Program (HITAP) plays a central role in policy and reimbursement decisions (Health Intervention and Technology Assessment Program (HITAP), 2021). However, no study to date has evaluated the economic value of HZ vaccination in patients with ESRD in Thailand, representing a critical evidence gap for equitable and cost-effective vaccine policy development.

Patients with ESRD represent a high-risk population for HZ because of profound immune dysfunction, high infection-related morbidity, and frequent exposure to healthcare settings (Betjes, 2013). Notably, the burden of both ESRD and HZ is greatest among older adults, in whom the incidence and severity of HZ – and the risk of complications such as post-herpetic neuralgia – increase substantially (Kuo et al., 2012). From an equity and disease-severity perspective, ESRD patients therefore constitute a particularly vulnerable group for whom economic evidence is essential to inform fair and cost-effective vaccine reimbursement decisions.

Building on our previous cost-utility analysis (CUA) of HZ vaccination in Thai adults living with Human Immunodeficiency Virus (HIV) (Kulthanachairojana et al., 2025) (another immunocompromised high-risk group), this study extends the evaluation of HZ vaccines to patients with ESRD, who face distinct clinical vulnerabilities and operational challenges in care delivery. In line with the international policy context and to address the lack of local evidence, we performed a model-based economic evaluation comparing ZVL, RZV, and no vaccination in patients with ESRD. The assessment included CUA and budget impact analysis (BIA) based on local epidemiological and cost data and aimed to generate robust evidence to inform policy making with regard to zoster vaccine administration in this high-risk population.

## Methods

CUA was conducted from a societal perspective, incorporating direct medical and direct non-medical costs, whereas BIA was performed from a payer perspective over a 5**-**year time horizon, in line with Thai health technology assessment (HTA) guidelines (Health Intervention and Technology Assessment Program (HITAP), 2021).

A model-based economic evaluation was conducted to compare the cost-effectiveness of three vaccination strategies for preventing HZ in patients with ESRD: (1) no vaccination, representing the current standard of care in Thailand, where HZ vaccines are not provided since they are not reimbursed under the Universal Coverage Scheme; (2) vaccination with a single dose of ZVL; and (3) vaccination with two doses of RZV. The first of these three served as the reference strategy, whereas the two vaccine-based alternatives were evaluated in terms of their incremental clinical and economic value.

The study protocol was reviewed and approved by the Institutional Review Board of Burapha University, Thailand (approval number IRB1-049/2567). All data inputs were sourced from publicly available literature, national datasets, or expert opinion, and no data from individual patients were collected.

### Model overview

A state-transition Markov model was constructed to evaluate the long-term clinical and economic outcomes of HZ vaccination in patients with ESRD. The model structure was adapted from a previously published framework developed for assessing HZ vaccination in Thai individuals living with HIV (Kulthanachairojana et al., 2025), with modifications to reflect the epidemiology and clinical course of ESRD. The hypothetical cohort represented Thai patients with ESRD aged 60 years **–** corresponding to the average age of patients with this disease (Satirapoj et al., 2025) – and was followed over a lifetime horizon.

**Figure 1.**
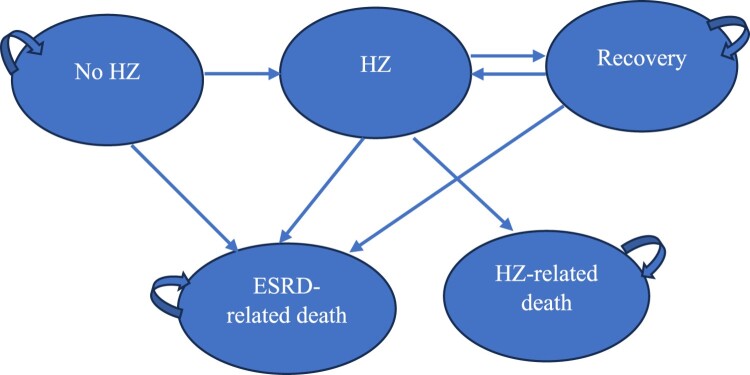
Markov model. Abbreviations: HZ, herpes zoster; ESRD, end-stage renal disease.

The model consisted of five mutually exclusive health states: no herpes zoster (no HZ), HZ infection, recovery, ESRD-related death, and HZ-related death, adapted from a previously published modelling framework (Kulthanachairojana et al., 2025). At model entry, all individuals were assumed to be in the no HZ state. During each annual cycle, individuals in this state could either remain without HZ, develop HZ infection, or die from ESRD-related causes. Patients who developed HZ entered the HZ infection state, where they could experience HZ-related complications. Following the acute phase, individuals either transitioned to the recovery state or died from HZ-related or ESRD-related causes. Patients in the recovery state were assumed to have resolved acute HZ symptoms but remained at risk of HZ recurrence or ESRD-related death in subsequent cycles. Both ESRD-related death and HZ-related death were modelled as absorbing states. A cycle length of one year was applied to reflect the natural history of HZ, the reporting of epidemiological inputs, and the annual pattern of vaccine effectiveness and waning. Half-cycle correction was applied to account for the timing of transitions occurring, on average, midway through each cycle. The overall model structure and allowable transitions between health states are illustrated in Figure 1.

Model validation included internal checks of model logic and numerical stability, as well as conceptual validation through expert consultation to ensure clinical plausibility in the Thai ESRD context.

### Epidemiological parameters

Due to the absence of Thailand-specific epidemiological data on HZ incidence among patients with ESRD, a model-based extrapolation approach was adopted. The epidemiological parameters incorporated into the model were derived from reviewed literature and reflected the most relevant data available for the ESRD population examined. A targeted literature review of peer-reviewed studies published between 2010 and 2025 was conducted using major biomedical databases, focusing on studies reporting HZ incidence, complications, recurrence, or mortality in ESRD or clinically comparable immunocompromised populations. Studies were screened based on predefined criteria, including relevance to the target population, consistency with standard clinical definitions of HZ outcomes, and applicability to healthcare settings comparable to that of Thailand.

The incidence of HZ was estimated by applying a relative risk adjustment to age-matched data from the general population to account for the increased susceptibility observed in dialysis patients (Kuo et al., 2012). The probabilities of HZ-related complications – including neurological and ophthalmic complications as well as disseminated infection and PHN – were calculated based on an international cohort study focusing specifically on ESRD patients (Yanni et al., 2018). The risk of HZ recurrence was modelled as an annual probability stratified by age, based on data from immunocompromised populations (Kim et al., 2018; Muñoz-Quiles et al., 2020), which were considered clinically relevant proxies in the absence of ESRD-specific recurrence data. HZ-related mortality was estimated using evidence from a targeted study examining the risk of mortality following HZ infection in patients with ESRD (Ahn et al., 2019). In contrast, baseline all-cause mortality among patients with ESRD was parameterised using Thai-specific survival estimates derived from a national cohort study, thereby reflecting local dialysis outcomes in Thailand (Changsirikulchai et al., 2018). When multiple parameter estimates were available, values were selected based on clinical plausibility and consistency across sources, and parameter uncertainty was incorporated through sensitivity analyses. Parameter values and plausible ranges are presented in Table 1. Based on the applied incidence and complication probabilities, the model implicitly estimates the annual number of HZ and PHN cases among patients with ESRD in Thailand.

**Table 1.** Model parameters.

Parameter	Value	Range	Distribution	Reference
Epidemiology				
Probability of transition from No HZ to HZ	0.07334	0.01225–0.0848	Beta	(Kuo et al., 2012)
Probability of neurological complication from HZ (other than PHN)	0.0056	0.0054–0.0077	Beta	(Yanni et al., 2018)
Probability of HZO	0.0288	0.0176–0.0288	Beta	(Yanni et al., 2018)
Probability of disseminated complication from HZ	0.0006	0.0003–0.0010	Beta	(Yanni et al., 2018)
Probability of PHN complication from HZ	0.1208	0.1024–0.1208	Beta	(Yanni et al., 2018)
Probability of Death from HZ	0.02322	0.01867–0.02816	Beta	(Ahn et al., 2019)
Probability of HZ recurrence yearly				
- Age 60–69	0.0116	0.0091–0.0148	–	(Muñoz-Quiles et al., 2020)
- Age 70–79	0.0132	0.0103–0.0169	–	(Muñoz-Quiles et al., 2020)
- Age 80 and more	0.0134	0.0105–0.0172	–	(Muñoz-Quiles et al., 2020)
Average Age of ESRD patients	60.0000	–	–	(Satirapoj et al., 2025)
Probability of death in patients with ESRD				
- 1 Year after ESRD	0.1740	–	–	(Changsirikulchai et al., 2018)
- 2 Years after ESRD	0.1308	–	–	(Changsirikulchai et al., 2018)
- 3 Years after ESRD	0.1086	–	–	(Changsirikulchai et al., 2018)
- 4 Years after ESRD	0.0859	–	–	(Changsirikulchai et al., 2018)
- 5 Years after ESRD and more	0.0769	–	–	(Changsirikulchai et al., 2018)
Vaccine First-dose coverage	0.6800	0.5220–0.6800	Beta	(Martino et al., 2023; Thewjitcharoen et al., 2020)
Second-dose compliance of RZV	1.0000	60.10–88.60	–	(Suranartwatchawong, 2022)
Vaccine efficacy				
- RZV efficacy 1–5 years after vaccination	0.8660	0.8660–0.9970	Beta	(Williams et al., 2025)
- RZV efficacy 6–7 years after vaccination	0.8850	0.7490–0.9560	Beta	(Williams et al., 2025)
- RZV efficacy 8–9 years after vaccination	0.8420	0.6790–0.9310	Beta	(Williams et al., 2025)
- RZV efficacy 10 years after vaccination	0.7320	0.4690–0.8760	Beta	(Williams et al., 2025)
- RZV efficacy for reducing PHN	0.9120	0.7590–0.9770	Beta	(Xia et al., 2022)
- RZV efficacy for reducing HZO and other complications	0.6700	0.6200–0.7100	Beta	(Xia et al., 2022)
- RZV efficacy reduction from baseline in case of one dose administration (first year)	20%	–	–	(George et al., 2025)
- RZ Efficacy reduction yearly in case of one dose administration (subsequent year)	18.20%	–	–	(George et al., 2025)
- ZVL efficacy 1 year after vaccination	0.6870	0.6630–0.7090	–	(Ansaldi et al., 2016)
- ZVL efficacy 2 years after vaccination	0.4950	0.4570–0.5310	–	(Ansaldi et al., 2016)
- ZVL efficacy 3–6 years after vaccination	0.3290	0.3290–0.3910	–	(Ansaldi et al., 2016)
- ZVL efficacy 7 years after vaccination	0.1650	0.0140–0.2930	–	(Ansaldi et al., 2016)
- ZVL efficacy 8 years after vaccination	0.0420	0.0420–0.2590	–	(Ansaldi et al., 2016)
- ZVL efficacy for reducing PHN	0.6650	0.4750–0.7920	–	(Xia et al., 2022)
- ZVL efficacy for reducing HZO and other complications	0.6300	0.3900–0.7700	–	(Xia et al., 2022)
- Probability of local/general AEs of RZV	0.003563	0.00371–0.00401	–	(Nelson et al., 2023)
- Probability of SAEs of RZV	0.00183	0.00173–0.00193	–	(Nelson et al., 2023)
- Probability of AEs local/general of ZVL	0.0010125	0.0009992–0.0010259	–	(Miller et al., 2018)
- Probability of SAEs of ZVL	0.0000445	0.0000417–0.0000473	–	(Miller et al., 2018)
Utility				
- ESRD patient baseline utility	0.83	0.81–0.83	Beta	(Rattanachotphanit et al., 2018)
Disutility			Beta	
- HZ	0.117	0.105–0.129	Beta	(Eriksson et al., 2020)
- PHN	0.186	0.167–0.205	Beta	(Eriksson et al., 2020)
- Other complications of HZ	−80%	(−80%)–(−84%)	Beta	(Sollie et al., 2022)
- Vaccine local/general AEs	0.0001	0.00009–0.00011	Beta	(Le & Rothberg, 2015)
-Vaccine SAEs	0.0082	0.00738–0.00902	Beta	(Le & Rothberg, 2015)
Direct medical costs (USD)			Beta	
- ZVL vaccine (1 dose)	61.61	49.29–73.93	Gamma	(Thai Red Cross AIDS and Infectious Diseases Research Centre (Anonymous Clinic), 2025)
- RZV vaccine (1 dose)	160.18	128.15–192.22	Gamma	(Thai Red Cross AIDS and Infectious Diseases Research Centre (Anonymous Clinic), 2025)
- Vaccine Local/general AEs treatment	0.42	0.34–0.50	Gamma	(Luangasanatip et al., 2021)
- Vaccine SAEs treatment	213.1	170.48–255.72	Gamma	(Luangasanatip et al., 2021)
- HZ treatment	119.78	119.78–315.83	Gamma	(Aunhachoke et al., 2011)
- HZ neurological complication treatment	382.46	382.46–1,969.54	Gamma	(Thai CaseMix Centre Health Systems Research Institute, 2024)
- HZO	236.36	236.36–897.88	Gamma	(Thai CaseMix Centre Health Systems Research Institute, 2024)
- HZ disseminated complication treatment	712.62	662.96–762.28	Gamma	(Thai CaseMix Centre Health Systems Research Institute, 2024)
- Vaccine administration	11.06	11.06–16.91	Gamma	(Health Intervention and Technology Assessment Program (HITAP), 2019)
- PHN treatment	246.44	154.02–308.04	Gamma	Expert opinion
Direct non-medical costs (USD)				
- Travelling cost	4.89	1.84–4.89	Gamma	(Riewpaiboon, 2012)
- Additional food cost	1.8	0.46–1.8	Gamma	(Riewpaiboon, 2012)
Parameters for budget impact analysis				
- Prevalence of ESRD in Thailand	110,764	–	–	(Satirapoj et al., 2025)
- Incidence of ESRD in Thailand	17,204	–	–	(Satirapoj et al., 2025)

Footnote: For parameters without reported uncertainty, ranges were assumed as ±20% of the base-case values.

Abbreviations: AE, adverse event; BIA, budget impact analysis; CUA, cost-utility analysis; ESRD, end-stage renal disease; HZ, herpes zoster; HZO, herpes zoster ophthalmicus; PHN, post-herpetic neuralgia; RZV, recombinant zoster vaccine; SAE, serious adverse event; USD, United States dollar; ZVL, zoster vaccine live.

### Vaccine-related parameters

The effectiveness of RZV and ZVL in preventing HZ was modelled using a time-dependent waning function, whereby vaccine efficacy declines over successive years following administration. Efficacy values at different time points were informed by long-term studies evaluating immunogenicity and clinical effectiveness (Ansaldi et al., 2016; Williams et al., 2025). In addition to overall HZ prevention, vaccine efficacy against specific complications was incorporated into the model based on data from a previous systematic review (Xia et al., 2022). To account for vaccine-related harms, the model also included the probability of adverse events (AEs). Estimates for local/general and serious AEs were derived from post-licensure surveillance data, reflecting real-world safety profiles for RZV and ZVL (Miller et al., 2018; Nelson et al., 2023).

### Cost inputs

CUA included direct medical and direct non-medical costs. Thai Baht (THB) to US dollars (USD) at an exchange rate of 1 USD = 32.4628 THB. All costs were analyzed and reported primarily in USD to ensure internal consistency across model inputs, results, figures, and tables. Corresponding values in Thai Baht (THB) are additionally presented for policy interpretation of key cost outcomes. Direct medical costs included vaccine acquisition and administration, management of vaccine-related AEs, treatment of HZ, and HZ-related complications. Unit costs for the vaccines were obtained from the Thai Red Cross Society (Thai Red Cross AIDS and Infectious Diseases Research Centre (Anonymous Clinic), 2025), whereas administration costs were derived from national costs for health promotion and prevention services in 2019, using the cost of influenza vaccination in the elderly as a proxy (Health Intervention and Technology Assessment Program (HITAP), 2019). Costs associated with AEs were sourced from previous vaccine-related economic evaluations conducted in Thailand (Luangasanatip et al., 2021); the cost of treating acute HZ episodes was derived from a previous study of HZ treatment costs in Thailand (Aunhachoke et al., 2011); and the management costs of HZ-related complications were estimated based on the Thai Diagnosis-Related Group system (Thai CaseMix Centre Health Systems Research Institute, 2024). Because no published data were available on the cost of PHN treatment in Thailand, a plausible estimate was obtained from expert opinion. The estimate was informed by structured input from clinicians with experience in managing herpes zoster and postherpetic neuralgia in Thai patients, reflecting routine clinical practice, including outpatient visits, pharmacological treatment, and follow-up care. Direct non-medical costs, including patient and caregiver transportation and meal expenses, were also included, based on the standard cost list for economic evaluations in Thailand (Riewpaiboon, 2012). Informal caregiver time and opportunity costs were not incorporated due to the absence of reliable Thailand-specific data, and to avoid speculative estimation beyond available empirical evidence.

### Utility inputs

The model outcome consisted in quality-adjusted life years (QALYs). Baseline utility values for patients with ESRD were derived from a Thai study assessing health-related quality of life among individuals receiving dialysis (Rattanachotphanit et al., 2018). To reflect temporary reductions in quality of life, disutility weights were applied for acute HZ episodes, HZ-related complications, and PHN, based on published sources (Eriksson et al., 2020). In addition, disutility values associated with vaccine-related AEs were incorporated into the model using estimates from previously published literature (Le & Rothberg, 2015).

### Cost-utility analysis

The cost-effectiveness ratio (ICER) was calculated as the difference in total costs divided by the difference in total QALYs between competing strategies. Each vaccination strategy – ZVL and RZV – was independently compared to the no-vaccination strategy. Future costs and QALYs were discounted at an annual rate of 3%, in accordance with Thai HTA guidelines (Health Intervention and Technology Assessment Program (HITAP), 2021). A strategy was cost-effective if its ICER fell below the Thai willingness-to-pay (WTP) threshold of USD 4,928.72 per QALY gained (160,000 THB/QALY). The model was constructed using TreeAge Pro 2025 software.

### Sensitivity analysis

#### One-way sensitivity analysis

A one-way sensitivity analysis was conducted to examine the impact of parameter uncertainty on the ICER. Key model inputs – including clinical probabilities, cost estimates, and utility values – were varied independently across predefined ranges to identify the most influential drivers of uncertainty. Parameter ranges were derived from published confidence intervals or reported variability where available. In the absence of empirical uncertainty estimates, plausible ranges (e.g. ±20% of base-case values) were applied. The results were summarised using a tornado diagram.

#### Probabilistic sensitivity analysis

A probabilistic sensitivity analysis (PSA) was performed to assess the joint impact of parameter uncertainty on model outcomes using Monte Carlo simulation with 1,000 iterations. Parameters supported by empirical uncertainty were assigned probability distributions according to standard health economic modelling practice, with beta distributions applied to probabilities and utility values and gamma distributions applied to cost parameters. The number of iterations was selected in accordance with ISPOR good modelling practices (Caro et al., 2012). Convergence was assessed by examining the stability of mean costs, mean QALYs, incremental outcomes, and the shape of cost-effectiveness acceptability curves (CEAC) across increasing numbers of simulations. Additional iterations did not materially change the estimated ICERs or the probability of cost-effectiveness, indicating adequate convergence of the PSA results. PSA outcomes were presented using cost-effectiveness planes and cost-effectiveness CEACs, illustrating the probability of each strategy being cost-effective across a range of WTP thresholds.

#### Scenario analysis

A scenario analysis was conducted to assess the impact of reduced adherence to the second dose of RZV. In contrast to the base-case model, which assumed full compliance with the two-dose schedule, this analysis considered two alternative scenarios at lower second-dose completion rates to reflect real-world adherence observed in clinical practice (Suranartwatchawong, 2022). This analysis aimed to evaluate how deviations from the ideal dosing regimen would influence the cost-effectiveness of the RZV strategy.

#### Budget impact analysis

Budget impact analysis (BIA) was conducted to estimate the financial implications of introducing HZ vaccination with ZVL and RZV among patients with ESRD from the perspective of a national healthcare payer in Thailand over a 5-year time horizon, in accordance with Thai HTA guidelines (Health Intervention and Technology Assessment Program (HITAP), 2021). Consistent with these guidelines, only direct medical costs were included, and future costs were not discounted. The analysis incorporated prevalent ESRD patients in the first year, with incident cases added annually in subsequent years. Epidemiological inputs were obtained from the Thailand Renal Replacement Therapy Registry, which provides nationally aggregated data on dialysis-treated ESRD patients and is considered representative of the treated ESRD population in Thailand (Satirapoj et al., 2025).

To reflect different implementation contexts, two vaccine uptake scenarios were evaluated. The base-case scenario assumed 100% vaccine uptake, whereby all prevalent ESRD patients would receive vaccination in the first year, and all newly diagnosed ESRD patients would be vaccinated upon initiation of dialysis thereafter. This scenario was intentionally designed to represent an upper-bound (worst-case) estimate of fiscal exposure, consistent with the primary objective of BIA to inform payers of the maximum potential budgetary commitment associated with introducing a new intervention. In addition, a moderate uptake scenario was explored to better reflect plausible real-world implementation. The moderate uptake rate was set at 52.2%, based on reported influenza vaccination uptake among patients with diabetes mellitus in Thailand (Thewjitcharoen et al., 2020). This scenario was used as a proxy for phased programme roll-out, accounting for real-world constraints such as procurement timelines, facility-level budget limitations, and regional variation in adoption.

## Results

### Cost-utility analysis

#### Base-case analysis

The lifetime costs and QALYs associated with each vaccination strategy are summarised in Table 2. In the base-case analysis, the no-vaccination strategy resulted in a total cost of USD 95.19 (THB 3,090.13) and yielded 5.6621 QALYs. ZVL and RZV vaccination strategies increased costs (USD 132.45 [THB 4,299.70] and USD 294.34 [THB 9,555.10], respectively) and improved health outcomes (5.6854 and 5.7303 QALYs, respectively), with the latter yielding the highest values. The ZVL and RZV strategies resulted in ICER values of USD 1,599.14 (THB 51,912.56) and USD 2,920.09 (THB 94,794.30) per QALY gained, respectively. As both values fall below the Thai WTP threshold, both vaccination strategies were deemed cost-effective in the base-case scenario. The component determining the higher cost of vaccination was the vaccine acquisition cost, calculated at USD 41.89 (THB 1,359.87) for ZVL and USD 217.84 (THB 7,071.70) for RZV. However, these additional costs were partially offset by reductions in HZ infection and its complications.

**Table 2.** Cost-utility analysis.

Components	No vaccine	ZVL	RZV
*Cost per patient (USD)*			
Direct medical cost			
- Vaccine	–	41.89	217.84
- Vaccine administration	–	7.52	15.04
- Vaccine AEs treatment	–	0.01	0.27
- HZ treatment	48.71	41.21	23.3
- HZ complication treatment	15.87	7.95	5.12
Direct non-medical cost	30.77	33.91	32.44
*Total cost (USD [THB])*	95.19 (3,090.13)	132.45 (4,299.70)	294.34 (9,555.10)
*QALYs*	5.6621	5.6854	5.7303
*ICER (USD/QALY [THB/QALY])*	Reference	1,599.14 (51,912.56)	2,920.09 (94,794.30)

Abbreviations: AE, adverse event; HZ, herpes zoster; ICER, incremental cost-effectiveness ratio; QALY, quality-adjusted life year; RZV, recombinant zoster vaccine; USD, United States dollar; ZVL, zoster vaccine live; THB, Thai Baht.

Footnote: Costs are presented in US dollars (USD) as the primary analytic currency. Corresponding values in Thai Baht (THB) are shown in parentheses for policy interpretation. Currency conversion was performed using an exchange rate of 1 USD = 32.4628 THB, with all costs standardised to the year 2025.

In addition to cost-effectiveness outcomes, the model estimated the clinical impact of herpes zoster vaccination in terms of health events avoided over the model time horizon. Compared with no vaccination, ZVL vaccination was associated with reductions of 0.0890 herpes zoster (HZ) cases, 0.0427 postherpetic neuralgia (PHN) cases, and 0.0021 HZ-related deaths per patient, reflecting expected lifetime outcomes in a normalised cohort of one. The RZV vaccination strategy yielded substantially greater clinical benefits, preventing 0.3577 HZ cases, 0.0575 PHN cases, and 0.0083 HZ-related deaths per patient relative to no vaccination. This corresponds to approximately 358 HZ cases, 58 PHN cases, and 8 HZ-related deaths prevented per 1,000 patients.

### Sensitivity analysis

#### One-way sensitivity analysis

**Figure 2.**
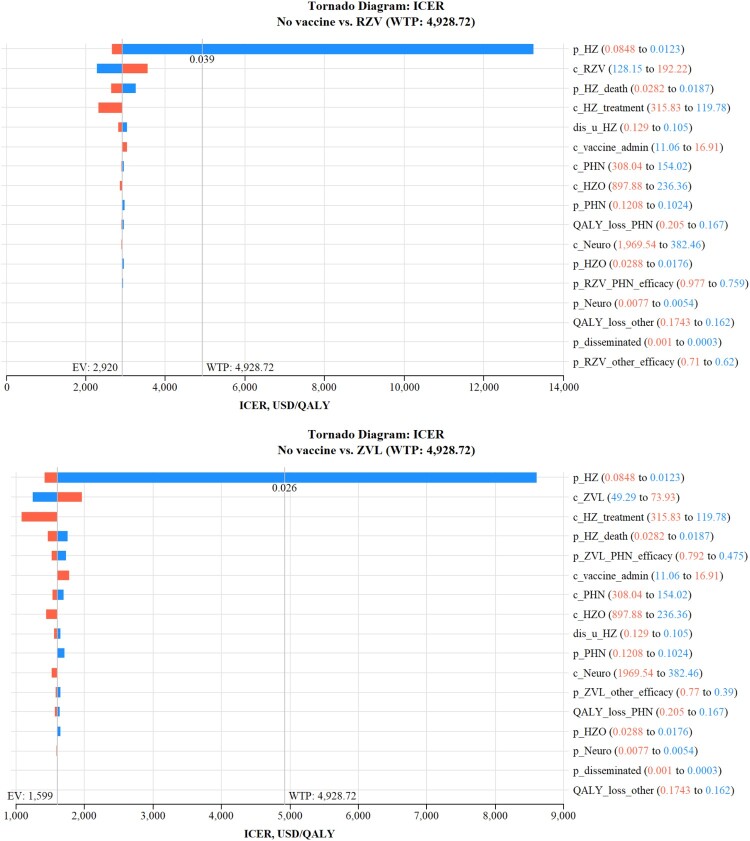
One-way sensitivity analysis. Abbreviations: HZ, herpes zoster; HZO, herpes zoster ophthalmicus; ICER, incremental cost-effectiveness ratio; PHN, post-herpetic neuralgia; QALY, quality-adjusted life year; RZV, recombinant zoster vaccine; USD, United States dollar; WTP, willingness-to-pay (threshold): ZVL, zoster vaccine live.

The results of one-way deterministic sensitivity analysis are presented in tornado diagrams in Figure 2. When comparing ZVL and no vaccination, the factors with the greatest influence on ICER were the probability of HZ infection, the cost of acquiring the ZVL vaccine, and the cost of treating acute HZ. Similarly, in the comparison between RZV and no vaccination, ICER was most sensitive to the probability of HZ infection, the cost of RZV, and the risk of HZ-related mortality. For ZVL, ICER exceeded the WTP threshold of USD 4,928.72 per QALY (160,000 THB/QALY) when the annual probability of HZ infection fell below 0.026. For RZV, the threshold exceeded was 0.039.

#### Probabilistic sensitivity analysis

**Figure 3.**
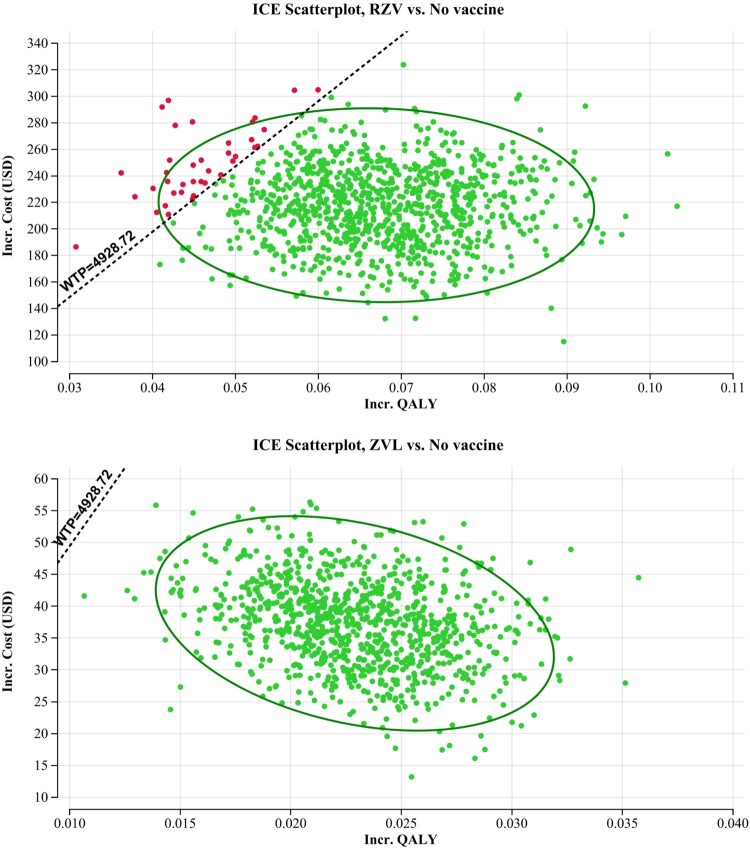
Probabilistic sensitivity analysis. Abbreviations: HZ, herpes zoster; ICER, incremental cost-effectiveness ratio; QALY, quality-adjusted life year; RZV, recombinant zoster vaccine; USD, United States dollar; ZVL, zoster vaccine live.

**Figure 4.**
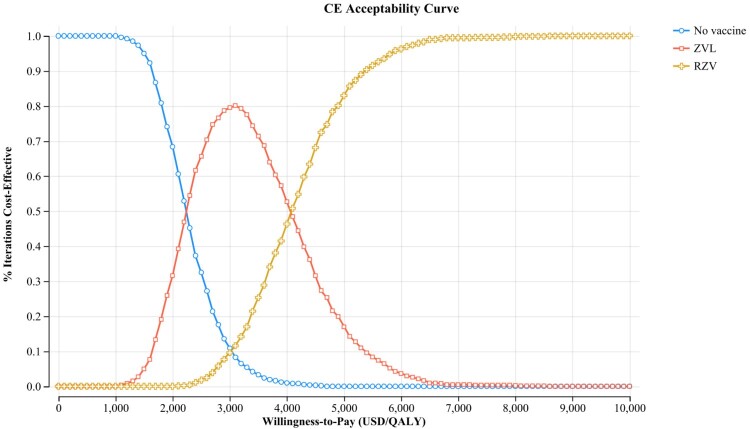
Cost-effectiveness acceptability curve. Abbreviations: CEAC, cost-effectiveness acceptability curve; QALY, quality-adjusted life year; RZV, recombinant zoster vaccine; USD, United States dollar; ZVL, zoster vaccine live.

The results of the PSA are illustrated in the cost-effectiveness scatterplots and CEAC included in Figures 3 and 4, respectively. When comparing each vaccination strategy to no vaccination, in all simulations for ZVL (100%) and in the majority of simulations for RZV (87.6%), ICER values fell below the Thai WTP threshold. Based on the CEAC, ZVL has the highest probability of being the optimal strategy at the Thai WTP threshold. In contrast, RZV surpasses ZVL in terms of the probability of cost-effectiveness only when the WTP threshold is increased substantially.

#### Scenario analysis

Scenario analyses were conducted to determine the influence of reduced compliance to the second dose of RZV on cost-effectiveness. While base-case analysis assumed full adherence (100%), two alternative scenarios considered lower compliance rates of 88.6% and 60.1%, reflecting actual adherence in the real world. When considering RZV versus no vaccination, at 88.6% compliance, ICER increased modestly to USD 2,961.27 (THB 96,128.87) per QALY gained, and at 60.1% compliance, it rose to USD 3,094.63 (THB 100,457.22) per QALY gained. Both values remained well below the Thai threshold.

#### Budget impact analysis:

The results of the 5-year BIA are presented in Table 3. Under the no-vaccination scenario, the projected cumulative healthcare expenditure was USD 6.93 million (THB 224.99 million). Assuming full vaccine uptake as an upper-bound scenario, implementation of ZVL vaccination resulted in a total 5-year budget of USD 16.48 million (THB 535.08 million), corresponding to an incremental budget impact of USD 9.55 million (THB 310.02 million) compared with no vaccination. In contrast, adoption of RZV was associated with substantially higher budgetary requirements, with a total 5-year budget of USD 62.35 million (THB 2,023.91 million) and an incremental cost of USD 55.42 million (THB 1,799.09 million).

**Table 3.** Five-year budget impact analysis under different vaccine uptake scenarios (USD [THB], million).

Cost components	No vaccine (Baseline)	ZVL		RZV	
		100% uptake	Moderate uptake	100% uptake	Moderate uptake
Vaccine	0	11.06 (359.17)	5.77 (187.42)	57.53 (1,867.59)	30.03 (974.88)
Vaccine administration	0	1.99 (64.48)	1.04 (33.72)	3.97 (128.95)	2.07 (67.27)
Vaccine AEs treatment	0	0.00178 (0.06)	0.00093 (0.03)	0.07 (2.28)	0.04 (1.19)
HZ and HZ-related complications treatment	6.93 (225.02)	3.43 (111.38)	5.10 (165.66)	0.77 (25.08)	3.71 (120.58)
Net 5-year budget impact	6.93 (225.02)	16.48 (535.08)	11.92 (386.83)	62.35 (2,023.91)	35.85 (1,163.92)
Incremental vs No vaccine	Reference	9.55 (310.02)	4.99 (161.86)	55.42 (1,799.09)	28.92 (938.95)

Abbreviations: HZ, herpes zoster; RZV, recombinant zoster vaccine; USD, United States dollar; ZVL, zoster vaccine live; THB, Thai Baht.

Footnote: All costs are presented in US dollars (USD) as the primary analytic currency. For policy interpretation in the Thai context, corresponding values in Thai Baht (THB) are additionally shown in parentheses. Currency conversion was performed using an exchange rate of 1 USD = 32.4628 THB, with all costs standardised to the year 2025.

When a more plausible moderate uptake scenario (52.2%) was considered, the projected budget impact decreased proportionally. Under this scenario, the incremental 5-year budget impact was reduced to USD 4.99 million (THB 161.86 million) for ZVL and USD 28.92 million (THB 938.95 million) for RZV relative to no vaccination, while maintaining the overall budgetary pattern observed in the upper-bound analysis.

## Discussion

Our economic evaluation demonstrated that both zoster vaccines – ZVL and RZV – are cost-effective for preventing HZ in Thai patients with ESRD. While RZV was associated with the highest cost, it also provided the greatest health benefit, particularly in reducing HZ-related morbidity. Sensitivity analyses confirmed the robustness of these findings, and real-world adherence scenarios showed sustained cost-effectiveness, supporting the practical feasibility of vaccine integration into ESRD care.

While several studies have examined the cost-effectiveness of HZ vaccination in immunocompromised groups – particularly among hematopoietic stem cell transplant and solid organ transplant recipients (George et al., 2025; Pedrazzoli et al., 2022; Salem et al., 2023) – economic evaluations focusing specifically on dialysis-dependent patients with ESRD remain limited. However, real-world data suggest that vaccination can reduce HZ incidence in this population (Tseng et al., 2016). Patients with ESRD are particularly vulnerable to HZ and associated complications because of immunological dysfunction brought on by the illness and long-term dialysis. Due to compromised immunity, the dormant varicella zoster virus can more readily reactivate and lead to HZ development. Moreover, PHN, a serious consequence of HZ infection, is frequent and burdensome for patients with ESRD (Ahn et al., 2019; Kuo et al., 2012).

Patients with ESRD are known to have profound immune dysfunction characterised by impaired cellular and humoral immune responses, chronic inflammation, and features of immunosenescence (Betjes, 2013). Evidence from studies of hepatitis B vaccination in patients with chronic kidney disease, including those receiving dialysis, indicates suboptimal immunogenicity and variable vaccine responses, reflecting an underlying state of vaccine hyporesponsiveness in this population (Fabrizi et al., 2021). These observations suggest that the real-world effectiveness of HZ vaccines in ESRD patients may be attenuated relative to the trial efficacy observed in healthier populations, particularly among immunocompromised hosts (Lal et al., 2015). In the absence of ESRD-specific efficacy data for HZ vaccines, our model relied on the best available evidence from the general population and selected immunocompromised cohorts, consistent with prior economic evaluations.

In this study, vaccine efficacy and waning assumptions were therefore derived from clinical trials conducted in the general population and selected immunocompromised groups, as no efficacy data specific to patients with ESRD are currently available. This approach may overestimate vaccine effectiveness in ESRD and consequently overestimate vaccine benefits while underestimating the ICER. However, it reflects a pragmatic modelling strategy commonly used in economic evaluations when disease-specific data are lacking. Importantly, our findings should be interpreted in light of real-world implementation considerations in dialysis settings, where fixed treatment schedules, vaccine reactogenicity, and logistical constraints may further influence vaccine uptake and effectiveness. In addition to immunological considerations, the implementation of HZ vaccination in dialysis settings involves practical challenges that may influence real-world uptake and costs. Rigid hemodialysis schedules and cold-chain requirements may limit flexibility for vaccine delivery, while differences in dosing schedules and reactogenicity profiles between ZVL and RZV may affect patient acceptance and service delivery. These operational factors should be considered when interpreting the economic findings and planning vaccine implementation in dialysis centres in Thailand.

Our findings can also be interpreted in relation to our previous economic evaluation of herpes zoster vaccination in Thai adults living with HIV (Kulthanachairojana et al., 2025). Although both HIV and ESRD represent high-risk immunocompromised populations, patients with ESRD generally have greater disease severity and poorer baseline prognosis, which may influence cost-effectiveness patterns and highlight the importance of equity considerations in vaccine reimbursement decisions.

The results of our study provide new economic insights for a population at a high risk of HZ infection in a middle-income country, contributing to filling a critical gap in the current literature. Nonetheless, several limitations should be acknowledged. First, cost inputs for HZ treatment were derived from data from the general population, which may not fully capture the higher clinical complexity and healthcare resource use required for patients with ESRD. To address this uncertainty, sensitivity analyses using increased cost assumptions were conducted, and the results demonstrated that the main findings remained robust. Similarly, in the absence of Thailand-specific data on the cost of PHN management, expert opinion was used to inform cost estimates, reflecting routine clinical practice in the Thai setting.

Second, due to the lack of Thailand-specific epidemiological data on HZ incidence among patients with ESRD, a model-based extrapolation approach was applied using ESRD-specific incidence estimates from a large population-based study in Taiwan. Although patients with ESRD have a substantially higher baseline risk of HZ, dialysis populations in Thailand may differ in clinical characteristics and healthcare utilisation patterns, introducing uncertainty in absolute burden estimates. In addition, patients with ESRD frequently present with multiple comorbidities, such as diabetes mellitus and cardiovascular diseases, which may further influence immune function and susceptibility to HZ. While these comorbidities were not modelled explicitly, their effects are implicitly reflected in the population-level epidemiological parameters and baseline utility values applied in the model.

Third, assumptions regarding vaccine coverage were informed by data from dialysis centres in other countries and by influenza vaccine uptake rates reported among Thai patients with chronic conditions. Moreover, the model did not account for potential regional variation in immune response or disparities in healthcare access, which may limit the generalizability of the findings. External validation against Thai longitudinal clinical or economic data was not feasible due to data limitations, a common challenge in modelling rare outcomes in specific patient subgroups. Furthermore, informal caregiver time and opportunity costs were not included due to the absence of reliable Thailand-specific data, which may result in an underestimation of the total societal burden.

Despite these limitations, they are more likely to affect absolute estimates of costs and health outcomes than the relative cost-effectiveness and budget impact comparisons across strategies, which are primarily driven by differences in intervention effects and costs. The robustness of the main conclusions was supported by deterministic and probabilistic sensitivity analyses conducted over plausible parameter ranges.

In line with the epidemiology of ESRD (Kuo et al., 2012), this study focused on patients aged 60 and above because ESRD is more prevalent in this older population cohort, and these patients are at the highest risk of developing both HZ and its severe complications. Dialysis-dependent patients often have immune dysfunction, which may increase susceptibility to other infections and influence recovery (Betjes, 2013). Vaccine selection should consider not only cost-effectiveness but also clinical suitability. ZVL may have limited applicability in individuals with a severely compromised immune status. In contrast, RZV is applicable to a broader range of ESRD patients and maintains high efficacy in preventing HZ-related complications (Advisory Committee on Immunization Practices, 2024; Marra et al., 2024). ZVL is administered as a single dose, whereas RZV requires two doses; however, neither is yet strongly recommended in Thailand’s national policy for this patient group, in contrast to policies in other countries. PHN is common in ESRD and poses a considerable burden as it is associated with lower pain tolerance, lower quality of life, and limited pain management options under renal drug dosing restrictions (Kitala-Tańska et al., 2024).

Preventing PHN is therefore of high clinical importance. Reducing HZ incidence in ESRD patients may also help avoid interruptions to dialysis services, decrease infection control workload, and lower the risk of nosocomial transmission in dialysis units (Centers for Disease Control and Prevention, 2023; Kumar et al., 2024). These considerations highlight the need for policies supporting cost-effective, clinically appropriate, and practical vaccination strategies. Such strategies may include phased introduction in high-volume dialysis centres and pilot implementation to assess feasibility and generate local evidence on vaccine effectiveness. Additional approaches may involve targeted price negotiations or inclusion in benefit packages for high-risk groups.

## Conclusion

This study demonstrates that herpes zoster vaccination using either ZVL or RZV is a cost-effective strategy for preventing HZ in Thai patients with ESRD, with RZV providing greater health benefits at higher cost. These conclusions should be interpreted with caution due to uncertainties in model inputs, particularly the use of non-ESRD data. These findings support prioritising HZ vaccination among patients with ESRD as a high-risk group, with consideration of pilot implementation to assess feasibility and system-level impact, given the substantial budget implications. Such pilot implementation may also help generate local evidence on vaccine effectiveness in the Thai ESRD population.

## Data Availability

All data are incorporated into the article.

## References

[CIT0001] Advisory Committee on Immunization Practices. (2024). *Evidence to recommendations framework for use of recombinant zoster vaccine in immunocompromised adults aged* ≥*19 years*. Centers for Disease Control and Prevention. Retrieved 8 August from https://www.cdc.gov/acip/evidence-to-recommendations/recombinant-zoster-immunocompromised-etr.html.

[CIT0002] Ahn, J. H., Waller, J. L., Baer, S. L., Colombo, R. E., Kheda, M. F., Nahman, N. S., Jr., & Turrentine, J. E. (2019). Mortality risk after herpes zoster infection in end-stage renal disease patients. *Clinical Kidney Journal*, *12*(1), 101–105. 30746135 10.1093/ckj/sfy058 PMC6366125

[CIT0003] Ansaldi, F., Trucchi, C., Alicino, C., Paganino, C., Orsi, A., & Icardi, G. (2016). Real-world effectiveness and safety of a live-attenuated herpes zoster vaccine: A comprehensive review. *Advances in Therapy*, *33*(7), 1094–1104. 27262452 10.1007/s12325-016-0355-0 PMC4939147

[CIT0004] Aunhachoke, K., Bussaratid, V., Chirachanakul, P., Chua-Intra, B., Dhitavat, J., Jaisathaporn, K., Kaewkungwal, J., Kampirapap, K., Khuhaprema, T., & Pairayayutakul, K. (2011). Measuring herpes zoster, zoster-associated pain, post-herpetic neuralgia-associated loss of quality of life, and healthcare utilization and costs in Thailand. *International Journal of Dermatology*, *50*(4), 428–435. 21413953 10.1111/j.1365-4632.2010.04715.x

[CIT0005] Betjes, M. G. (2013). Immune cell dysfunction and inflammation in end-stage renal disease. *Nature Reviews Nephrology*, *9*(5), 255–265. 10.1038/nrneph.2013.44 23507826

[CIT0006] Caro, J. J., Briggs, A. H., Siebert, U., & Kuntz, K. M. (2012). Modeling good research practices – overview: A report of the ISPOR-SMDM modeling good research practices task force-1. *Value in Health*, *15*(6), 796–803. 10.1016/j.jval.2012.06.012 22999128

[CIT0007] Centers for Disease Control and Prevention. (2023). *Preventing Varicella-Zoster Virus (VZV) transmission from herpes zoster in healthcare settings*. Centers for Disease Control and Prevention. Retrieved August 8, from https://www.cdc.gov/shingles/hcp/infection-control/index.html.

[CIT0008] Changsirikulchai, S., Sriprach, S., Thokanit, N. S., Janma, J., Chuengsaman, P., & Sirivongs, D. (2018). Survival analysis and associated factors in Thai patients on peritoneal dialysis under the PD-first policy. *Peritoneal Dialysis International*, *38*(3), 172–178. 29437140 10.3747/pdi.2017.00127

[CIT0009] Drolet, M., Brisson, M., Schmader, K. E., Levin, M. J., Johnson, R., Oxman, M. N., Patrick, D., Blanchette, C., & Mansi, J. A. (2010). The impact of herpes zoster and postherpetic neuralgia on health-related quality of life: A prospective study. *Canadian Medical Association Journal*, *182*(16), 1731–1736. 20921251 10.1503/cmaj.091711 PMC2972323

[CIT0010] Eriksson, J., Hunger, M., Bourhis, F., Thorén, R., Popmihajlov, Z., Finelli, L., & Jiang, Y. (2020). Cost and utility in immunocompromised subjects who developed herpes zoster during the randomized V212 inactivated varicella-zoster vaccine (ZVIN) trial. *Expert Review of Pharmacoeconomics & Outcomes Research*, *20*(6), 613–621. 31721601 10.1080/14737167.2020.1693267

[CIT0011] Fabrizi, F., Cerutti, R., Dixit, V., & Ridruejo, E. (2021). Hepatitis B virus vaccine and chronic kidney disease. The advances. *Nefrologia (Engl Ed)*, *41*(2), 115–122. 10.1016/j.nefro.2020.08.016 36165374

[CIT0012] George, S., Carrico, J., Hicks, K. A., Loukov, D., Ng, C., & Curran, D. (2025). Cost-effectiveness and public health impact of recombinant zoster vaccine versus no herpes zoster vaccination in selected populations of immunocompromised adults in Canada. *BMC Health Services Research*, *25*(1), 604. 40281614 10.1186/s12913-025-12550-x PMC12023514

[CIT0013] Government of, C. (2022). *Process for incorporating economic evidence into federal vaccine recommendations: National Advisory Committee on Immunization (NACI)*. https://www.nitag-resource.org/sites/default/files/2022-08/Process%20for%20incorporating%20economic%20evidence%20into%20federal%20vaccine%20recommendations_%20National%20Advisory%20Committee%20on%20Immunization%20%28NACI%29%20-%20Canada.ca_.pdf.

[CIT0014] Health Intervention and Technology Assessment Program (HITAP). (2019). *Cost Analysis of health promotion and disease prevention services (Fiscal year 2019)*. Health Intervention and Technology Assessment Program; supported by NHSO (Thailand). Retrieved August 8, from https://www.hitap.net/wp-content/uploads/2020/06/full-report-Cost-PP.pdf.

[CIT0015] Health Intervention and Technology Assessment Program (HITAP). (2021). *Guideline for health technology assessment in Thailand (2021)*. Ministry of Public Health.

[CIT0016] Kim, Y. J., Lee, C. N., Lee, M. S., Lee, J. H., Lee, J. Y., Han, K., & Park, Y. M. (2018). Recurrence rate of herpes zoster and its risk factors: A population-based cohort study. *Journal of Korean Medical Science*, *34*(2), e1. 30636941 10.3346/jkms.2019.34.e1 PMC6327089

[CIT0017] Kitala-Tańska, K., Kania-Zimnicka, E., Tański, D., Kwella, N., Stompór, T., & Stompór, M. (2024). Prevalence and management of chronic pain, including neuropathic pain, in dialysis patients with end-stage renal disease. *Medical Science Monitor: International Medical Journal of Experimental and Clinical Research*, *30*, e943808–943801. 38751083 10.12659/MSM.943808 PMC11107387

[CIT0018] Kulthanachairojana, N., Hemapanpairoa, J., Santhaveesook, C., Piboonsatsanasakul, P., & Fueymee, A. (2025). Cost-utility and budget impact analyses of herpes zoster vaccines in patients with human immunodeficiency virus in Thailand. *Value in Health Regional Issues*, *48*, 101119. 40315778 10.1016/j.vhri.2025.101119

[CIT0019] Kumar, D., Jorga, A., Yun, H., & Williams, C. (2024). # 2434 literature review on the burden of herpes zoster in patients with kidney disease – a need for earlier prevention. *Nephrology Dialysis Transplantation*, *39*(Supplement_1), gfae069-0524-2434.

[CIT0020] Kuo, C. C., Lee, C. T., Lee, I. M., Ho, S. C., & Yang, C. Y. (2012). Risk of herpes zoster in patients treated with long-term hemodialysis: A matched cohort study. *American Journal of Kidney Diseases*, *59*(3), 428–433. 10.1053/j.ajkd.2011.10.049 22178678

[CIT0021] Lal, H., Cunningham, A. L., Godeaux, O., Chlibek, R., Diez-Domingo, J., Hwang, S. J., Levin, M. J., McElhaney, J. E., Poder, A., Puig-Barberà, J., Vesikari, T., Watanabe, D., Weckx, L., Zahaf, T., & Heineman, T. C. (2015). Efficacy of an adjuvanted herpes zoster subunit vaccine in older adults. *New England Journal of Medicine*, *372*(22), 2087–2096. 10.1056/NEJMoa1501184 25916341

[CIT0022] Le, P., & Rothberg, M. B. (2015). Cost-effectiveness of herpes zoster vaccine for persons aged 50 years. *Annals of Internal Medicine*, *163*(7), 489–497. 26344036 10.7326/M15-0093

[CIT0023] Lim, D. Z. J., Tey, H. L., Salada, B. M. A., Oon, J. E. L., Seah, E.-J. D., Chandran, N. S., & Pan, J. Y. (2024). Herpes zoster and post-herpetic neuralgia – diagnosis, treatment, and vaccination strategies. *Pathogens*, *13*(7), 596. 39057822 10.3390/pathogens13070596 PMC11280284

[CIT0024] Luangasanatip, N., Mahikul, W., Poovorawan, K., Cooper, B. S., Lubell, Y., White, L. J., Teerawattananon, Y., & Pan-Ngum, W. (2021). Cost-effectiveness and budget impact analyses for the prioritisation of the four available rotavirus vaccines in the national immunisation programme in Thailand. *Vaccine*, *39*(9), 1402–1414. 33531197 10.1016/j.vaccine.2021.01.051

[CIT0025] Marra, F., Yip, M., Cragg, J. J., & Vadlamudi, N. K. (2024). Systematic review and meta-analysis of recombinant herpes zoster vaccine in immunocompromised populations. *PLoS One*, *19*(11), e0313889. 10.1371/journal.pone.0313889 39585863 PMC11588208

[CIT0026] Martino, F. K., Pini, S., Scaparrotta, G., Schirinzi, M., Gnappi, M., Fragasso, A., Zanella, R., Naso, E., De Giorgi, M. L., & Carraro, G. (2023). Recombinant varicella zoster vaccine in haemodialysis facilities: Adherence and safety. *Journal of Nephrology*, *36*(7), 2155–2158. 37392330 10.1007/s40620-023-01690-0

[CIT0027] Matanock, A., Lee, G., Gierke, R., Kobayashi, M., Leidner, A., & Pilishvili, T. (2020). Advisory Committee on Immunization Practices (ACIP) Evidence to Recommendations (EtR) framework. *MMWR. Morbidity and Mortality Weekly Report*, *69*(6), 165–168. 10.15585/mmwr.mm6906a4

[CIT0028] Miller, E. R., Lewis, P., Shimabukuro, T. T., Su, J., Moro, P., Woo, E. J., Jankosky, C., & Cano, M. (2018). Post-licensure safety surveillance of zoster vaccine live (Zostavax®) in the United States, Vaccine Adverse Event Reporting System (VAERS), 2006–2015. *Human Vaccines & Immunotherapeutics*, *14*(8), 1963–1969. 29580194 10.1080/21645515.2018.1456598 PMC6150039

[CIT0029] Muñoz-Quiles, C., López-Lacort, M., Díez-Domingo, J., & Orrico-Sánchez, A. (2020). Herpes zoster risk and burden of disease in immunocompromised populations: A population-based study using health system integrated databases, 2009–2014. *BMC Infectious Diseases*, *20*(1), 905. 33256624 10.1186/s12879-020-05648-6 PMC7708196

[CIT0030] Nelson, J. C., Ulloa-Pérez, E., Yu, O., Cook, A. J., Jackson, M. L., Belongia, E. A., Daley, M. F., Harpaz, R., Kharbanda, E. O., & Klein, N. P. (2023). Active postlicensure safety surveillance for recombinant zoster vaccine using electronic health record data. *American Journal of Epidemiology*, *192*(2), 205–216. 36193854 10.1093/aje/kwac170 PMC9896469

[CIT0031] Pedrazzoli, P., Lasagna, A., Cassaniti, I., Ferrari, A., Bergami, F., Silvestris, N., Sapuppo, E., Di Maio, M., Cinieri, S., & Baldanti, F. (2022). Vaccination for herpes zoster in patients with solid tumors: A position paper on the behalf of the Associazione Italiana di Oncologia Medica (AIOM). *ESMO Open*, *7*(4), 100548. 35853350 10.1016/j.esmoop.2022.100548 PMC9434335

[CIT0032] Public Health, E. (2013). *Code of practice for the Joint Committee on Vaccination and Immunisation (JCVI)*. https://assets.publishing.service.gov.uk/government/uploads/system/uploads/attachment_data/file/224864/JCVI_Code_of_Practice_revision_2013_-_final.pdf.

[CIT0033] Rattanachotphanit, T., Waleekhachonloet, O., Chanasopon, S., Ausornsagiam, W., Kanjanasilp, J., & Suwattanasilp, A. (2018). Quality of life and utilities of end stage renal disease patients undergoing dialysis. *Isan J Pharm Sci*, *14*(4), 88–98.

[CIT0034] Riewpaiboon, A. (2012). PRM3 standard cost list for economic evaluation in Thailand. *Value in Health*, *15*(7), A645. 24964710

[CIT0035] Saguil, A., Kane, S., Mercado, M., & Lauters, R. (2017). Herpes zoster and postherpetic neuralgia: Prevention and management. *American Family Physician*, *96*(10), 656–663. 29431387

[CIT0036] Salem, A., La, E. M., Curran, D., Patterson, B. J., Carrico, J., Lorenc, S., Hicks, K. A., Poston, S., & Carpenter, C. F. (2023). Cost-effectiveness of recombinant zoster vaccine for the prevention of herpes zoster in hematopoietic stem cell transplant recipients and other immunocompromised adults in the United States. *PharmacoEconomics-Open*, *7*(6), 975–985. 37917310 10.1007/s41669-023-00438-7 PMC10721768

[CIT0037] Satirapoj, B., Tantiyavarong, P., Thimachai, P., Chuasuwan, A., Lumpaopong, A., Kanjanabuch, T., & Ophascharoensuk, V. (2025). Thailand renal replacement therapy registry 2023: Epidemiological insights into dialysis trends and challenges. *Therapeutic Apheresis and Dialysis*, *29*(5), 721–729. 40523870 10.1111/1744-9987.70056

[CIT0038] Sollie, M., Jepsen, P., & Sørensen, J. A. (2022). Patient-reported quality of life in patients suffering from acute herpes zoster – a systematic review with meta-analysis. *British Journal of Pain*, *16*(4), 404–419. 36032345 10.1177/20494637211073050 PMC9411760

[CIT0039] Suranartwatchawong, S. S. (2022). Implementation of personal vaccination record (PVR) to increase completeness and adherence of multi-dose adult vaccination: A retrospective study at the wellness center, Burapha University Hospital. *Journal of Nakornping Hospital*, *13*(1), 117–127.

[CIT0040] Thai CaseMix Centre Health Systems Research Institute. (2024). *Thai Diagnosis Related Group (TDRG), version 6.3: Grouping manual (Vols. 1–2, with appendices F1 and F2)*. Thai CaseMix Centre. Retrieved August 8, from https://www.tcmc.or.th/download-tcmc.

[CIT0041] Thai Red Cross AIDS and Infectious Diseases Research Centre (Anonymous Clinic). (2025). *Vaccination service*. Retrieved August 8, from https://th.aidsid.or.th/vaccination-service/.

[CIT0042] Thewjitcharoen, Y., Butadej, S., Malidaeng, A., Yenseung, N., Nakasatien, S., Lekpittaya, N., Kittipoom, W., Krittiyawong, S., & Himathongkam, T. (2020). Trends in influenza and pneumococcal vaccine coverage in Thai patients with type 2 diabetes mellitus 2010–2018: Experience from a tertiary diabetes center in Bangkok. *Journal of Clinical & Translational Endocrinology*, *20*, 100227. 32395432 10.1016/j.jcte.2020.100227 PMC7212954

[CIT0043] Tseng, H. F., Luo, Y., Shi, J., Sy, L. S., Tartof, S. Y., Sim, J. J., Hechter, R. C., & Jacobsen, S. J. (2016). Effectiveness of herpes zoster vaccine in patients 60 years and older with end-stage renal disease. *Clinical Infectious Diseases*, *62*(4), 462–467. 26671505 10.1093/cid/civ930

[CIT0044] Williams, L. R., Hombach, J., & Marti, M. (2025). Evaluating the immunogenicity, efficacy, and effectiveness of recombinant zoster vaccine for global public health policy. *Vaccines*, *13*(3), 250. 40266145 10.3390/vaccines13030250 PMC11946835

[CIT0045] World Health, O. (2024). *Strategic Advisory Group of Experts (SAGE) on immunization: 2024 meeting highlights*. https://cdn.who.int/media/docs/default-source/immunization/sage/2024/september/sage-sept_2024-highlights_final.pdf.

[CIT0046] Xia, Y., Zhang, X., Zhang, L., & Fu, C. (2022). Efficacy, effectiveness, and safety of herpes zoster vaccine in the immunocompetent and immunocompromised subjects: A systematic review and network meta-analysis. *Frontiers in Immunology*, *13*, 978203. 36248796 10.3389/fimmu.2022.978203 PMC9561817

[CIT0047] Yanni, E. A., Ferreira, G., Guennec, M., El Hahi, Y., El Ghachi, A., Haguinet, F., Espie, E., & Bianco, V. (2018). Burden of herpes zoster in 16 selected immunocompromised populations in England: A cohort study in the clinical practice research datalink 2000–2012. *BMJ Open*, *8*(6), e020528. 10.1136/bmjopen-2017-020528 PMC6009512 29880565

[CIT0048] Yawn, B. P., & Gilden, D. (2013). The global epidemiology of herpes zoster. *Neurology*, *81*(10), 928–930. 23999562 10.1212/WNL.0b013e3182a3516e PMC3885217

